# Dietary Diversity and Associated Factors Among Children in the Tahtay Maichew District, Northern Ethiopia

**DOI:** 10.1155/jnme/2092071

**Published:** 2026-05-18

**Authors:** Ermyas Brhane Reda, Teklit Grum, Kiros Gereziher Arefayne, Ebud Ayele Dagnazgi, Teklehaymanot Huluf Abraha, Gebrekiros Aregawi, Shewit Engdashet Berhe

**Affiliations:** ^1^ Department of Human Nutrition, Aksum University, Aksum, Ethiopia, aku.edu.et; ^2^ Department of Reproductive Health, Aksum University, Aksum, Ethiopia, aku.edu.et; ^3^ Department of Midwifery, Aksum University, Aksum, Ethiopia, aku.edu.et

**Keywords:** children 6–23 months, dietary diversity, Ethiopia, factors associated, Tahtay Maichew district

## Abstract

**Background:**

Children aged 6–23 months are in a period of intense physical and neurological development and therefore require nutrient‐dense complementary foods from multiple food groups. However, information on what young children eat and which factors influence the variety of their diets remains scarce in the study area. Therefore, this study was conducted to assess the level of dietary diversity among 6–23‐month‐old children in the Tahtay Maichew district and to identify demographic, household, and health‐service–related determinants of their food variety.

**Methods:**

A community‐based cross‐sectional study was carried out among 981 children aged 6–23 months in the Tahtay Maichew district, northern Ethiopia. Dietary diversity was evaluated using a 24‐h maternal recall of all foods and beverages consumed by the child. After checking questionnaires for completeness and consistency, data were coded and entered into EPI INFO Version 7 and then exported to STATA‐12 for analysis. In the final multivariable model, variables with a *p* value < 0.05, identified through backward elimination, were considered factors associated with dietary diversity.

**Results:**

Only 31.1% (95% CI: 28.3%–34.3%) of children received the recommended minimum dietary diversity for their age. Having a housewife mother (AOR = 1.76, 95% CI = 1.15–2.7), being from a government‐employed mother (AOR = 2.67, 95% CI = 1.03–6.87), having a father with primary (AOR = 1.67, 95% CI = 1.01–2.77) or secondary education (AOR = 2.12, 95% CI = 1.26–3.57), attending postnatal care follow‐up (AOR = 1.54, 95% CI = 1.08–2.21), and good maternal knowledge of child feeding were significantly associated with achieving recommended dietary diversity among children.

**Conclusion:**

The proportion of children meeting the minimum dietary diversity was low. Mothers’ occupation, husband’s educational status, postnatal care follow‐up, and maternal knowledge of child feeding were independent determinants of dietary diversity.

## 1. Background

Children aged 6–23 months are in a period of intense physical and neurological development and therefore require nutrient‐dense complementary foods from multiple food groups. Inadequate quality of complementary foods at this stage is a major contributor to undernutrition, growth faltering, micronutrient deficiencies, and child mortality in low‐ and middle‐income countries, including Ethiopia [1, 2]. Dietary diversity, measured as the number of WHO food groups consumed in the 24 h preceding the survey, is widely used as a proxy for micronutrient adequacy and overall diet quality in this age group [3–5].

Globally, less than one‐fourth of children 6–23 months of age receive the minimum recommended dietary diversity, and Ethiopia is among the countries with poor indicators [6, 7]. Nationally, fewer than one in five Ethiopian children achieve minimum dietary diversity; their diets rely heavily on staples such as cereals, roots, and tubers, with limited intake of fruits, vegetables, and animal‐source foods [1, 8–10]. This inadequate complementary feeding practice contributes to the high burden of stunting, underweight, micronutrient deficiency, and infections among children in the country [2, 11, 12]. Consequently, these various forms of malnutrition can lead to limited cognitive development, poor long‐term career prospects, and reduced productivity [13].

A range of socioeconomic, health service, and caregiver‐related factors are known to be linked to dietary diversity among children. Higher education of parents, urban residence, ownership of livestock or having home gardens, higher household wealth index, and better food security are positively associated with dietary diversity of children in multiple studies conducted in Ethiopia [3, 4, 7, 10, 14]. Maternal exposure to nutritional messages through mass media, receipt of counseling from health extension workers, and participation in cooking demonstrations and growth monitoring programs were also associated with diverse diets among children [3, 4, 7, 11, 15, 16]. Additionally, health service‐related factors, such as antenatal care (ANC), institutional delivery, and postnatal care (PNC), were associated with adequate child dietary diversity [1, 4, 7, 15, 16].

Although children’s health, growth, and development require adequate dietary diversity in early life, as noted above, information on what they eat and on the factors that influence the variety of their diets remains scarce in the study area. Context‐specific studies are crucial because there are variations in dietary patterns, agricultural production, access to services, and sociocultural norms across districts. Therefore, this study was conducted to assess the level of dietary diversity among 6–23‐month‐old children in the Tahtay Maichew district and to identify demographic, household, and health‐service–related determinants of their food variety.

## 2. Methods and Materials

### 2.1. Study Design and Setting

A community‐based cross‐sectional study was carried out among 981 children aged 6–23 months in the Tahtay Maichew district, northern Ethiopia. The data for this study were collected as part of a larger survey on Infant and Young Child Feeding Practices. The overall methodology and the preliminary results on meal frequency were initially shared as a preprint [17] and subsequently published in PLOS Global Public Health [18]. This specific analysis focuses on the dietary diversity component of the survey.

The recruitment period for this study was from 22/1/2018 to 30/3/2018. The Tahtay Maichew district is located in the central zone of the Tigray Regional State in Ethiopia, 1041 km from Addis Ababa, the capital city. A mid‐altitude climate characterizes the district and comprises 17 kebeles (the smallest administrative unit in Ethiopia).

### 2.2. Sample Size Determination

The sample size for this study was determined using the single‐population proportion formula. Calculations were performed for the two primary outcomes of the original survey: minimum meal frequency (MMF) and minimum dietary diversity (MDD). Based on prevalence estimates from the 2016 Ethiopian Demographic and Health Survey [8], the required sample sizes were 981 for MMF and 477 for MDD. To ensure adequate statistical power, the larger sample size of 981 was adopted. Further details of the calculation are provided in the previously published study [18].

### 2.3. Sampling Procedure

A multistage sampling method was employed to select child‐mother pairs. Initially, eight kebeles were selected through simple random sampling. The total sample size was then allocated proportionally across the selected kebeles. A rapid census was conducted to identify households with children aged 6–23 months. Finally, eligible child‐mother pairs were selected from each kebele. The detailed sampling procedure has been previously described in our related work on meal frequency [18].

### 2.4. Measurements

Data were collected from mothers of children aged 6–23 months using a structured, interviewer‐administered questionnaire adapted from previous studies [1, 8, 9, 11, 14, 19–25]. The questionnaire was developed in English, translated into Tigrigna (the local language), and back‐translated to ensure consistency. Data were collected using the Tigrigna version of the questionnaire by eight data collectors and two supervisors, all of whom were fluent in the local language.

Dietary diversity was assessed using a single 24‐h recall, in which mothers reported all foods and beverages their child had consumed in the preceding day. These items were categorized into seven standard food groups. Children were considered to have met the minimum dietary diversity if they had consumed foods from at least four of these seven groups in the previous 24 h [26, 27].

The household wealth index was estimated using data on 24 asset variables, each coded as either 0 (not owned) or 1 (owned). Then we examined the suitability of these variables for Principal Component Analysis (PCA) using the Kaiser–Meyer–Olkin (KMO) and communality values. The KMO threshold was 0.8, indicating sampling adequacy, while the communality values were 0.6, suggesting the extracted components well represent the variables. After that, components with eigenvalues greater than 1 were retained, as this indicates they explain more variance. Then, factor scores were generated using PCA. Finally, these factor scores were summed and used to classify households into three wealth categories: poor, medium, and rich [11].

Mothers’ knowledge of complementary feeding was assessed using child‐feeding‐related questions. Those who scored above the mean were classified as having good knowledge [28]. Similarly, maternal social capital was assessed using six questions; respondents who scored above the mean were considered to have good social capital [29]. Additionally, media exposure was operationalized as mothers’ reports of how often they engaged with three traditional channels: print news (newspaper or magazine), radio, and television. Women were classified as having satisfactory media exposure if they reported using any of these media at least once per week [9, 11].

### 2.5. Data Processing and Analysis

After checking questionnaires for completeness and consistency, data were coded and entered into EPI INFO version 7 and then exported to STATA‐12 for analysis. Continuous variables were summarized using means and standard deviations, while categorical variables were described using frequencies and percentages. Normality of data was checked using the skewness test and P‐P plot.

Initially, associations between each explanatory variable and dietary diversity were explored using simple logistic regression. Covariates with a bivariate *p* value ≤ 0.05 were included in a multivariable logistic regression model. Model adequacy was checked with the Hosmer–Lemeshow goodness‐of‐fit test, and collinearity was evaluated using variance inflation factors (< 10) and tolerance values (> 0.1). A backward stepwise procedure was applied, and predictors retaining a *p* value < 0.05 in the final model were deemed independently associated with dietary diversity. Adjusted odds ratios with 95% confidence intervals were used to quantify the magnitude of these associations.

### 2.6. Ethics Approval and Consent to Participate

Before starting the study, ethical approval was obtained from the Institutional Review Board of the College of Health Sciences at Aksum University (Reference number: IRB 044/2018). Also, a letter of permission was received from the Tahtay Maichew district health office. Written informed consent was obtained from the mothers, and the information collected was kept anonymous.

## 3. Results

### 3.1. Sociodemographic and Socioeconomic Characteristics of Study Participants

A total of 949 mother‐child pairs provided complete responses, yielding a response rate of 96.7%. The mean age of the children was 13.7 ± 4.59 months, and the mean maternal age was 29.74 ± 6.66 years. The average family size was 4.75 ± 1.7 persons. Most mothers were married (89.4%) and identified as Orthodox Christian (92.5%). More than half (55.4%) were farmers, and 34.9% had no formal education (Table 1).

**TABLE 1:** Socioeconomic characteristics of participants in the Tahtay Maichew district, northern Ethiopia.

Variable	Category	Achieved minimum dietary diversity	
		Yes (%)	No (%)
Mother’s age in years	15–24	78 (8.2)	158 (16.6)
	25–34	149 (15.7)	318 (33.5)
	≥ 35	68 (7.1)	178 (18.9)

Age of the index child in months	6–11	98 (10.3)	261 (27.5)
	12–17	118 (12.4)	237 (25)
	18–23	79 (8.3)	156 (16.5)

Sex of the index child	Male	160 (16.9)	326 (34.4)
	Female	135 (14.2)	328 (34.5)

Family size in persons	2–4	166 (17.5)	329 (34.7)
	5–7	112 (11.8)	264 (27.8)
	≥ 8	17 (1.8)	61 (6.4)

Religion	Orthodox	258 (27.2)	620 (65.3)
	Muslim	37 (4)	34 (3.5)

Marital status of mother	Married	272 (28.7)	576 (60.7)
	Divorced	16 (1.7)	60 (6.4)
	Separated	3 (0.3)	5 (0.5)
	Widowed	4 (0.4)	13 (1.4)

Mother’s educational status	No formal education	55 (5.8)	276 (29.1)
	Primary school (1–8)	96 (10.1)	221 (23.3)
	Secondary school (9–12)	110 (11.6)	142 (15)
	Diploma and above	34 (3.5)	15 (1.6)

Husband’s educational status	No formal education	29 (3.4)	153 (17.7)
	Primary school (1–8)	96 (11.1)	244 (28.2)
	Secondary school (9–12)	107 (12.4)	159 (18.4)
	Diploma and above	44 (5)	33 (3.8)

Mother’s employment status	Farmer	122 (12.9)	404 (42.6)
	House wife	100 (10.5)	144 (15.2)
	Self employed	30 (3.2)	40 (4.2)
	Daily worker	8 (0.8)	47 (4.9)
	Government employed	35 (3.7)	19 (2)

Husband’s employment status	Farmer	147 (17)	440 (50.7)
	Self employed	57 (6.6)	47 (5.5)
	Daily worker	28 (3.3)	67 (7.7)
	Government employed	44 (5.1)	35 (4.1)

Wealth index	Low	73 (7.7)	244 (25.7)
	Middle	99 (10.4)	224 (23.6)
	High	123 (13)	186 (19.6)

Mother’s knowledge of child feeding	Good	251 (26.5)	298 (31.4)
	Poor	44 (4.6)	356 (37.5)

Satisfactory media exposure	Yes	144 (15.2)	163 (17.2)
	No	151 (15.9)	491 (51.7)

Mother’s social capital	Good	198 (20.9)	270 (28.4)
	Poor	97 (10.2)	384 (40.5)

### 3.2. Reproductive and Health Service Utilization‐Related Characteristics

The mean parity of the mothers was 2.84 ± 1.67 children. Most mothers (90.2%) attended at least one ANC visit, and 83.5% delivered at a health facility. Less than half, 45.3%, received PNC, while 61.4% reported at least one growth monitoring visit for the index child (Table 2).

**TABLE 2:** Reproductive and health service utilization characteristics of participants in the Tahtay Maichew district, northern Ethiopia.

Variable	Category	Achieved minimum dietary diversity	
		Yes (%)	No (%)
ANC follow‐up	Yes	284 (29.9)	572 (60.3)
	No	11 (1.2)	82 (8.5)

Number of ANC follow‐ups	1–3	77 (9)	245 (28.6)
	≥ 4	207 (24.2)	327 (38.2)

Birth preparedness	Yes	276 (29.1)	545 (57.4)
	No	19 (2)	109 (11.5)

Place of delivery of the index child	Health facility	274 (28.9)	518 (54.6)
	Home	21 (2.2)	136 (14.3)

Birth order of the index child	First	83 (8.7)	172 (18.2)
	Second to fifth	195 (20.5)	418 (44.1)
	Sixth and above	17 (1.8)	64 (6.7)

Birth interval between the older and index child in months	12–36	70 (10.1)	172 (24.8)
	37–60	106 (15.3)	230 (33.1)
	≥ 61	37 (5.3)	79 (11.4)

PNC follow‐up	Yes	191 (20.1)	239 (25.2)
	No	104 (11)	415 (43.7)

Number of PNC follow‐ups	1‐2	165 (38.4)	223 (51.9)
	≥ 3	26 (6)	16 (3.7)

Counseling about child feeding during ANC or PNC follow‐up	Yes	210 (22.1)	272 (28.9)
	No	85 (9)	382 (40)

Current contraceptive use	Yes	210 (22.1)	392 (41.3)
	No	85 (9)	262 (27.6)

Growth monitoring follow‐up	Yes	225 (23.7)	358 (37.7)
	No	70 (7.4)	296 (31.2)

Counseling about child feeding during growth monitoring follow‐up	Yes	209 (35.8)	314 (53.9)
	No	16 (2.7)	44 (7.4)

HEW home visit and counseling about child feeding within a month	Yes	173 (18.2)	261 (27.5)
	No	122 (12.9)	393 (41.4)

Abbreviations: ANC, antenatal care; PNC, postnatal care.

### 3.3. Dietary Diversity and Food Group Consumption

**FIGURE 1:**
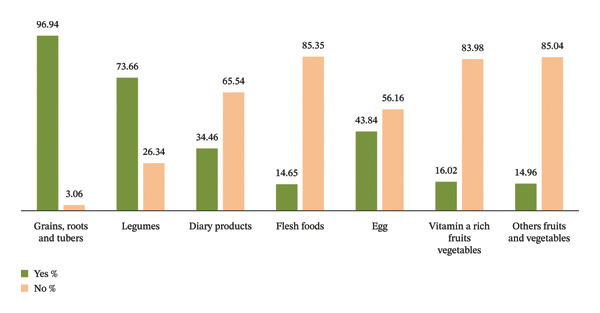
Food groups consumed by children aged 6–23 months, Tahtay Maichew district, northern Ethiopia.

Only 31.1% (95% CI: 28.3, 34.3%) of children received the recommended minimum dietary diversity for their age. Most of the children (96.94%) were fed grains, tubers, and roots, while only a small percentage (14.65%) consumed flesh foods (Figure 1).

### 3.4. Factors Associated With Minimum Dietary Diversity Among Children Aged 6–23 Months

In the bi‐variable logistic regression analysis, nine variables: wealth index, mother’s occupation, husband’s educational status, place of delivery, PNC follow‐up, health extension worker home visits, mother’s knowledge of child feeding, media exposure, and mother’s social capital were significantly associated with dietary diversity. However, in the multivariable logistic regression analysis, only four variables, namely, mother’s occupation, husband’s educational status, PNC follow‐up, and mother’s knowledge of child feeding, remained significantly associated with dietary diversity (Table 3).

**TABLE 3:** Factors associated with minimum dietary diversity among children aged 6–23 months, Tahtay Maichew district, northern Ethiopia.

Variable		Achieved minimum dietary diversity		COR with 95% CI	AOR with 95% CI
		Yes (%)	No (%)		
Wealth index	Poor	73 (23)	244 (77)	1	
	Middle	99 (30.7)	224 (69.3)	1.48 (1.04, 2.1)	1.02 (0.67, 1.55)
	Rich	123 (39.8)	186 (60.1)	2.21 (1.56, 3.13)	0.82 (0.49, 1.38)

Mother’s occupation	Farmer	126 (24)	400 (76)	1	
	Housewife	98 (40.2)	146 (59.8)	2.3 (1.66, 3.19)	1.76 (1.15, 2.7)^∗^
	Self‐employed	30 (42.9)	40 (57.1)	2.49 (1.48, 4.16)	1.42 (0.68, 2.94)
	Daily worker	8 (14.5)	47 (85.5)	0.56 (0.26, 1.22)	0.46 (0.15, 1.48)
	Government employed	33 (61.1)	21 (38.9)	6.1 (3.37, 11.05)	2.67 (1.03, 6.87)^∗^

Husband’s educational status	No formal education	27 (14.8)	155 (85.2)	1	
	Primary	100 (29.4)	240 (70.6)	2.06 (1.31, 3.29)	1.67 (1.01, 2.77)^∗^
	Secondary	107 (40.2)	159 (59.8)	3.55 (2.23, 5.66)	2.12 (1.26, 3.57)^∗^
	Diploma	18 (45)	22 (55)	4.77 (2.85, 9.97)	1.29 (0.51, 3.27)
	Degree and above	24 (64.9)	13 (35.1)	10.99 (4.97, 24.33)	2.71 (0.92, 7.97)

Place of delivery	Health facility	275 (34.7)	517 (65.3)	3.43 (2.11, 5.55)	1.49 (0.83, 2.56)
	Home	20 (12.7)	137 (87.3)	1	

PNC follow‐up	Yes	191 (44.4)	239 (55.6)	3.19 (2.39, 4.25)	1.54 (1.08, 2.21)^∗^
	No	104 (20)	415 (80)	1	

HEW home visit	Yes	176 (40.6)	258 (59.4)	2.14 (1.61, 2.82)	1.03 (0.73, 1.47)
	No	119 (23.1)	396 (76.9)	1	

Mother’s knowledge of child feeding	Good	251 (45.7)	298 (54.3)	7.81 (5.41, 11.27)	4.68 (3.05, 7.18)^∗^
	Poor	44 (11)	356 (89)	1	

Satisfactory media exposure	Yes	144 (46.9)	163 (53.1)	2.87 (2.15, 3.84)	1.31 (0.89, 1.94)
	No	151 (23.5)	491 (76.5)	1	

Mother’s social capital	Good	198 (42.3)	270 (57.4)	2.78 (2.08, 3.71)	1.23 (0.86, 1.77)
	Poor	97 (20.2)	384 (79.8)	1	

Abbreviations: AOR, adjusted odds ratio; COR, crude odds ratio.

^∗^ *p* value < 0.05.

Children with housewife mothers were 1.76 times more likely to receive minimum dietary diversity than children whose mothers were farmers (AOR = 1.76, 95% CI = 1.15–2.7). Similarly, children of government‐employed mothers were 2.67 times more likely to receive minimum dietary diversity than those of farmer mothers (AOR = 2.67, 95% CI = 1.03–6.87). Additionally, children whose fathers had primary education were 1.67 times more likely to achieve minimum dietary diversity than those whose fathers had no formal education (AOR = 1.67, 95% CI = 1.01–2.77). Likewise, children whose fathers had secondary education were 2.12 times more likely to receive minimum dietary diversity than those whose fathers had no formal education (AOR = 2.12, 95% CI = 1.26–3.57).

Children whose mothers had PNC follow‐up were 1.54 times more likely (AOR = 1.54, 95% CI = 1.08–2.21) to receive minimum dietary diversity than their counterparts. In addition, children of mothers with good knowledge of child feeding were 4.68 times more likely (AOR = 4.68, 95% CI = 3.05–7.18) to achieve the minimum dietary diversity than those whose mothers had poor knowledge.

## 4. Discussion

This study found that 31.1% (95% CI: 28.3%–34.3%) of children received the recommended dietary diversity for their age. Our previously published work on the same study population found a relatively higher proportion of children achieving the minimum meal frequency [18]. However, this study shows that only a small proportion of the children achieved minimum dietary diversity. This discrepancy indicates that caloric intake (quantity) is not being accompanied by nutrient density (quality).

Mother’s occupation, husband’s educational status, PNC follow‐up, and maternal knowledge of child feeding were significantly associated with dietary diversity among children. The proportion of children aged 6–23 months who received the minimum dietary diversity in this study was comparable to reports from Kenya, Tanzania, and Uganda (30%–40%) [19]. However, it was higher than findings from southern Ethiopia (27.3%) [14], western Ethiopia (23.7%) [30], and North West Ethiopia (17%) [11]. These discrepancies could be attributed to differences in sample size, study design, and underlying socioeconomic factors across settings.

Maternal employment status was also associated with children’s dietary diversity. Compared with children of farmer mothers, children of housewives and government‐employed mothers had 1.76‐and 2.67‐fold higher odds, respectively, of receiving the minimum dietary diversity. Multiple studies in Ethiopia and other low‐income countries support this finding [14, 31, 32]. This may be due to two factors. First, housewives might have enough time to prepare a variety of foods for their children. Second, employed mothers tend to have higher and more stable incomes, which enable them to buy diverse foods [32–34].

The odds of receiving minimum dietary diversity among children of fathers with primary education and secondary education were 1.67 and 2.12 times those of children whose fathers had no formal education, respectively. Multiple studies from other low and middle‐income countries, including Ethiopia, also reported similar findings [4, 28, 35, 36]. Educated husbands tend to have higher incomes and greater food security, better nutritional knowledge, and give greater support for their wives, thereby improving their children’s dietary diversity [35–37].

Children whose mothers attended PNC visits were 1.54 times more likely to receive minimum dietary diversity compared to their counterparts. Other studies from Ethiopia [7, 9, 11, 15] and Tanzania, another low‐income country [38], are in line with this. The finding implies that PNC services give a chance for behavioral change through counseling, which likely improves awareness, confidence, and motivation to provide diversified diets [11, 15, 16, 39].

Finally, maternal knowledge of child feeding was also a key predictor of children’s dietary diversity. Children of mothers with good knowledge of child feeding were 4.67 times more likely to achieve minimum dietary diversity than children of mothers with poor knowledge. This finding is consistent with other studies from Ethiopia [3, 28]. This may be because knowledge helps mothers understand the importance of a diverse diet for their children, rather than relying on staples [3, 40].

This study was community‐based, which enhances its external validity, despite being restricted to a small geographic area. However, its cross‐sectional design cannot establish causality due to temporal ambiguity. Moreover, the use of the single‐day 24‐h recall method to assess dietary diversity may have introduced recall and social desirability biases and is unlikely to capture usual day‐to‐day intake. Future studies could include a larger, more diverse sample to improve generalizability. In addition, longitudinal and mixed‐methods designs may better elucidate causal relationships and temporal changes.

## 5. Conclusion

According to WHO indicators, the proportion of children meeting the minimum dietary diversity was low. Factors significantly associated with dietary diversity included the mother’s occupation, husband’s educational level, PNC follow‐up, and maternal knowledge of child feeding. Health professionals and health extension workers should support mothers by providing timely and appropriate counseling on child feeding practices during PNC and growth‐monitoring follow‐up visits.

## Author Contributions

Ermyas Brhane Reda conceived the idea, developed the proposal, supervised data collection, and participated in data analysis and thesis writing. Teklit Grum, Kiros Gereziher Arefayne, Ebud Ayele Dagnazgi, Teklehaymanot Huluf Abraha, and Gebrekiros Aregawi contributed to proposal writing, data analysis, and interpretation. Shewit Engdashet Berhe was involved in proposal development, data collection supervision, analysis, thesis writing, and wrote the manuscript.

## Funding

This study was supported by a small‐scale research grant from Aksum University.

## Disclosure

All authors reviewed and approved the final manuscript.

## Conflicts of Interest

The authors declare no conflicts of interest.

## Data Availability

The datasets used during the current study are available from the corresponding author upon reasonable request.
